# Cutis verticis gyrata primitif essentiel, une affection cutanée rare: cas clinique et revue de la littérature

**DOI:** 10.11604/pamj.2014.19.345.5729

**Published:** 2014-12-03

**Authors:** Boukind Samira, Dlimi Meriem, Elatiqi Oumkeltoum, Elamrani Driss, Benchamkha Yassine, Ettalbi Saloua

**Affiliations:** 1 Service de Chirurgie Plastique, Réparatrice, Esthétique et Brûlés, CHU Mohammed VI, Marrakech, Maroc

**Keywords:** Cutis verticis gyrata, pachydermie, scalp, réduction de scalp, Cutis verticis gyrata, pachydermia, scalp, scalp reduction

## Abstract

Le cutis verticis gyrata (CVG), du cuir chevelu, est une maladie rare et évolutive de la peau du scalp. Elle est caractérisée par une hypertrophie et une hyperlaxité cutanée formant des plis semblables aux gyri du cortex cérébral. Nous présentons le cas d'une patiente de 21 ans atteinte de CVG primitif essentiel, ayant débuté à l’âge de 8 ans, au niveau du scalp et était d'aggravation progressive. La malade présentait une déformation du scalp avec de nombreux plis longitudinaux et transversaux. Sa demande était motivée par une gêne sociale et esthétique. Une résection chirurgicale de l'excédent cutané dans un plan transversal et longitudinal était réalisée. Le traitement de cette maladie est chirurgical, par l'excision des plis cutanés et remise en tension du scalp. Le nombre et la localisation des incisions doivent préserver la vascularisation des lambeaux de scalp et tenir compte du caractère évolutif de cette pathologie.

## Introduction

Le cutis verticis gyrata (CVG), ou pachydermie vorticellée du cuir chevelu, est une affection rare et évolutive de la peau du scalp et/ou du visage. Elle se caractérise par une hypertrophie et une hyperlaxité cutanée formant des plis semblables à la surface du cortex cérébral [1, 2]. Ces plis peuvent être à l'origine d'une gêne esthétique, sociale et fonctionnelle (macération, infection) [3]. Cette pathologie était rapportée pour la première fois par Alibert en 1837 sous le terme de « cutis sulcata » [3]. Robert était le premier ayant donné la description clinique de cette pathologie en 1843 [1] et en 1907, Unna lui donnait le nom de CVG, communément admis depuis [3]. Il existe trois formes décrites de CVG: **le CVG secondaire** à des pathologies chroniques métaboliques, inflammatoires [3], respiratoires, cardiaques, endocriniennes [4], hépatobiliaires et formes paranéoplasiques [1, 3], ou encore iatrogène suite à un traitement par Minoxidil [5]. Le CVG est également présent dans la pachydermoperiostose (ou ostéo-arthrite hypertrophique chronique) [6], les lésions hamartomateuses, l'acromégalie, la syphilis, la leucémie, la neurofibromatose, le syndrome d'Ehlers- Danlos [2]. Une traction chronique du scalp a même été décrite à l'origine d'un CVG [3]. Cette forme est la plus fréquente et touche plus fréquemment le visage en association avec l'atteinte du cuir chevelu. Elle intéresse surtout les hommes avec un sex-ratio de 5/1 [3]; **le CVG primitif**, beaucoup plus rare, dont l'origine est génétique à transmission indéterminée [3]. Il peut être essentiel ou non-essentiel: a forme non-essentielle est présente chez 0,5% des patients ayant des anomalies neurologiques et/ou ophtalmiques (retard mental, épilepsie, microcéphalie, encéphalopathies, cataracte congénitale) [1, 3]; la forme primitive essentielle est une forme extrêmement rare, seuls 15 cas de CVG primitif essentiel traités chirurgicalement sont rapportés dans la littérature. Elle touche surtout le sujet masculin et débute à la fin de l'adolescence. Cette forme n'est pas associée à des anomalies neurologiques ou ophtalmiques. Le type de transmission génétique est incertain [3].

## Patient et observation

**Figure 1:**
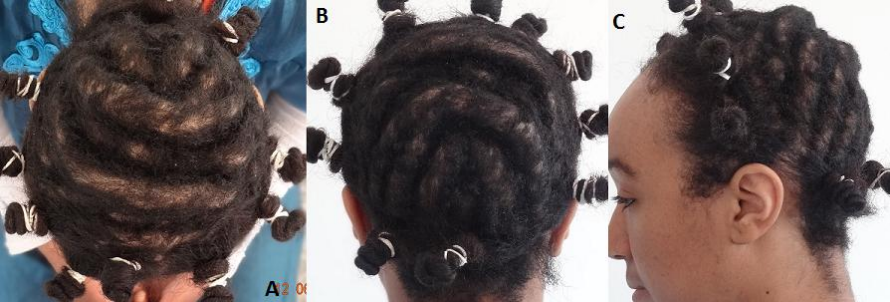
(A, B, C) hyperlaxité et hypertrophie du scalp avec sillons dans un axe coronal et sagittal. Vue de haut, postérieure, et profil gauche

**Figure 2:**
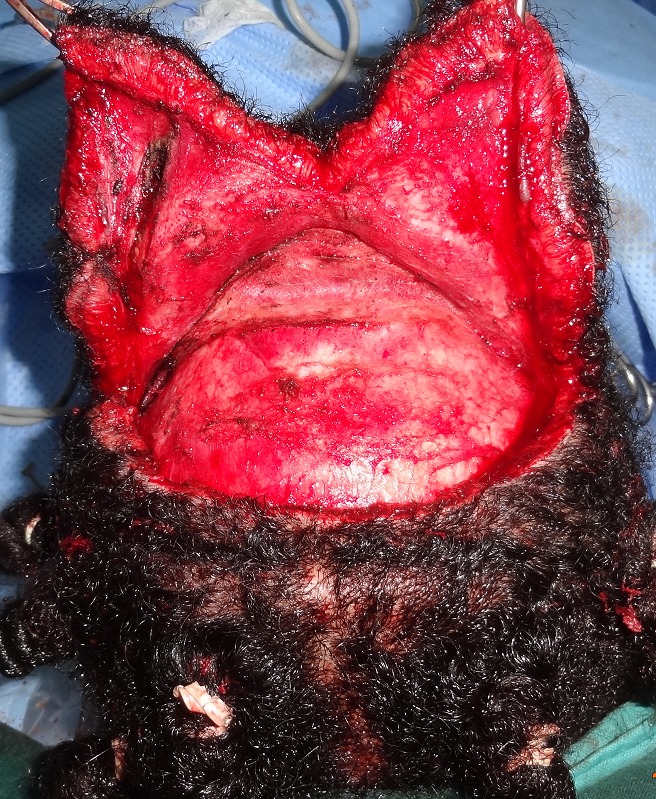
Photo après incision coronale sur le vertex et le scalp pariétal, complétée par une incision sagittale médiane sur le scalp occipital, puis décollement des lambeaux pariéto- occipitaux dans le plan sous galéal: nous avons remarqué sur ces lambeaux décollés une hyperplasie du tissu adipeux sous-cutané

Nous présentons le cas d'une patiente de 21 ans atteinte de CVG primitif essentiel. Le début de la maladie remonte à l’âge de 8 ans au niveau du scalp et était d'aggravation progressive. Aucun facteur déclenchant n’était identifié, ni de pathologie chronique sous jacente pouvant être à l'origine d'une forme secondaire de CVG. Par ailleurs la patiente présentait deux cas similaires dans la famille (sœur et tente paternelle). Seul le scalp était atteint sous la forme d'une hypertrophie et d'une hyperlaxité cutanée responsable d'un excès longitudinal et transversal, siégeant surtout au niveau de la région du vertex, occipitale haute et pariétale (Figure 1). La demande de la patiente était motivée par une gêne esthétique et sociale, à l'origine d'un complexe. À la palpation, ces plis semblaient formés d'une alternance de zones de peau d’épaisseur normale et de peau très épaisse. L'examen clinique et l'interrogatoire ne retrouvaient en outre aucun signe associé en dehors d'un prurit au niveau des lésions, aucun traitement en cours et les capacités intellectuelles de la patiente étaient normales. Une excision chirurgicale selon une incision coronale sur le vertex et le scalp pariétal, complétée par une incision sagittale médiane sur le scalp occipital, était réalisée suivie d'un décollement postérieur (Figure 2).

**Figure 3:**
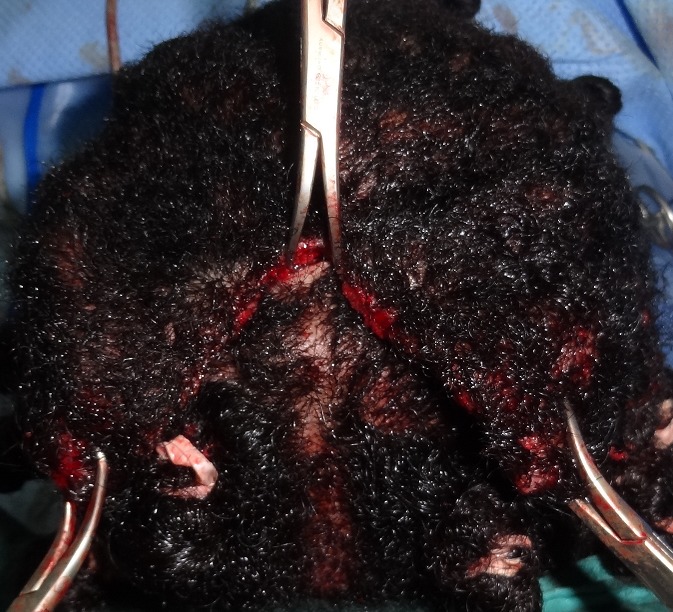
Les lambeaux sont avancés en avant pour être ramenés en tension jusqu'au niveau de la berge antérieure de l'incision coronale, afin de déterminer au mieux l'excédent cutané à réséquer

**Figure 4:**
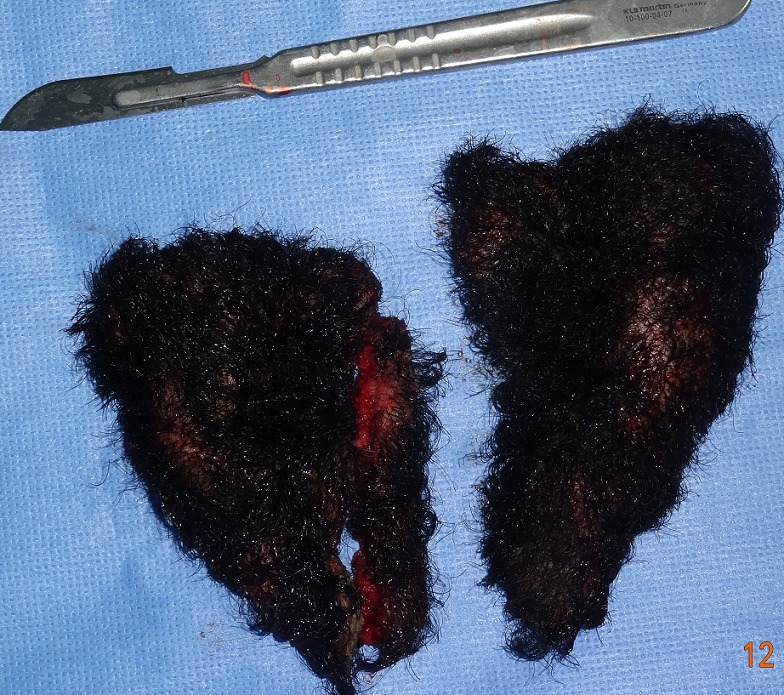
L'excédent cutané réséqué

**Figure 5:**
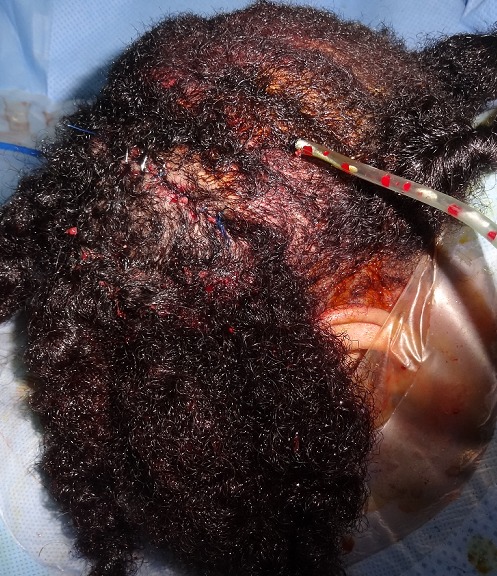
Photo finale après suture sur drain aspiratif

La patiente était installée en décubitus ventral sur têtière neurochirurgicale. L'intervention était menée sous anesthésie générale. Après infiltration du tissu sous cutané au sérum adrénaliné, la peau et la galéa étaient incisées selon les dessins pré- établis. Les lambeaux pariéto- occipitaux étaient ensuite décollés dans le plan sous-galéal (Figure 2) puis avancés en avant pour être ramenés en tension jusqu'au niveau de la berge antérieur de l'incision coronale, afin de déterminer au mieux l'excédent cutané à réséquer (Figure 3). Les pièces opératoires étaient conservées pour analyse anatomopathologique (Figure 4). La fermeture était effectuée en trois plans (galéal, dermique profond et superficiel), sur drain aspiratif (Figure 5). Les suites opératoires étaient simples, le drain était retiré à J1 du post opératoire et la patiente était déclarée sortante au deuxième jour post opératoire. Nous avons remarqué sur les lambeaux décollés et excisés une hyperplasie du tissu adipeux sous-cutané (Figure 2) qui était confirmée histologiquement. L’étude anatomopathologique parlait d'un épaississement fibreux des cloisons inter-lobulaires, un derme anormalement épais, et une hyperplasie des glandes sébacées. La patiente était satisfaite du résultat final.

## Discussion

Le CVG du cuir chevelu est une affection rare dont trois formes étiopathogéniques se distinguent: secondaire (la plus fréquente), primitive non essentielle et primitive essentielle. Notre patiente présentait une forme rare de CVG, dite primitive essentielle. Le caractère en commun entre ces trois formes est l'atteinte du scalp à type d'hypertrophie et d'hyperlaxité cutanée à l'origine de plis dans les deux axes, transversal et longitudinal. Les aires affectées au niveau du scalp peuvent être différents d'un individu à l'autre. Les lésions sont souvent développées au niveau du vertex, tandis que les régions temporale, frontale, et occipitale sont occasionnellement atteintes, avec possibilité d'atteinte de l'ensemble du scalp dans certains cas [7]. Le CVG ne concerne pas uniquement le scalp, mais d'autres localisations ont été décrites dans la littérature comme la face. Varun. H et al a rapporté un cas de CVG de la région glabellaire, la racine et le dorsum du nez [7]. L'aire atteinte est asymptomatique dans la majorité des cas. Le prurit était rapporté dans quelques cas de la littérature [7], ce qui est le cas chez notre patiente également. Les symptômes associés dépendent de la cause sous jacente dans le CVG secondaire [2]. Les trois formes du CVG sont lentement évolutives et ont un caractère récidivant après chirurgie. Un traitement médical par isotrétinoïne a été proposé mais s'est avéré de faible efficacité [3]. La chirurgie est le traitement morphologique de choix de cette affection, mais ne prévient pas de la poursuite évolutive de la maladie [3].

Certains auteurs ont préconisé une abstention thérapeutique le plus longtemps possible. La chirurgie semble, pour notre analyse, indiquée dès lors que le patient en fait la demande, qu'elle soit motivée par une gêne esthétique, psychologique ou fonctionnelle (macération, infection). Différentes techniques chirurgicales sont décrites, dont l'indication dépond de la sévérité, la localisation, la taille des lésions, la pathologie sous jacente et les souhaits du patient. Il peut s'agir d'une simple excision avec suture directe (pour les formes localisées de petite taille), la mise en place d'expandeurs cutanés au niveau du scalp sain et levée d'un lambeau d'avancement, ou une greffe cutanée immédiate après résection totale [2, 7], ou résection partielle des segments les plus hypertrophiques et les plus plissés [1, 3]. Des lambeaux musculo- cutanés ou lambeaux libres, comme le muscle grand dorsal, ont était également décrit pour des résections complètes des plis cutanés excédants [1]. Le schéma des excisions doit prendre en compte quatre éléments principaux: la position des cicatrices doit tenir compte du caractère évolutif de la maladie pour autoriser de futures excisions; le schéma d'excision doit absorber un excès dans les deux axes; le schéma doit pouvoir absorber lors de la première et des futures interventions l'excès cutané sur l'ensemble du scalp (frontal, pariéto-temporal et occipital); les incisions doivent créer des lambeaux à vascularisation fiable [3]. Nombreux sont les schémas d'excision qui ont été décrits dans la littérature; Kara rapporte le cas d'un patient traité suivant deux incisions bicoronales [3]. Al-Malaq et al. ainsi que Horch et al. décrivent des résultats similaires par excision directe des plis cutanés [3]. Radwanski et al. rapporte un schéma d'excision en « fleur de Lys », tenant compte ainsi des deux composantes de l'excès cutané [3]. Snyder décrit un cas traité par expansion cutanée, exérèse large et lambeaux de rotation [3]. Anuj. M et al rapporte le cas d'un patient dont le traitement a consisté en une expansion cutané avec levée d'un lambeau d'avancement du scalp [8]. Enfin, Misirlioglu et al. Rapporte le traitement d'un cas par excision circulaire et lambeaux de couverture en hélice [9]. Henrique N et al [1] ont conclut que la résection partielle avec suture directe est la technique la plus efficace et la plus sûre pour la cure du CVG. Etant donné que la maladie est cutanée (pachydermie) avec un respect de la galéa, il apparaît logique qu'une remise en tension du scalp par décollement dans l'espace de Merkel rend difficile la correction totale de l'affection. Pour pallier à ce problème, des incisions longitudinales de la galéa peuvent être pratiquées, en regard des plis résiduels, sans compromettre la vascularisation des lambeaux.

## Conclusion

Le CVG du cuir chevelu est une affection rare et évolutive responsable d'un handicap social et esthétique. Un bilan clinique et paraclinique sont primordiales pour éliminer la forme secondaire et primitive non essentielle. Le traitement de choix est la chirurgie par l'excision des zones cutanées les plus hypertrophiques et remise en tension du scalp dans le plan longitudinal et transversal. Les excisions doivent tenir compte de l'anatomie vasculaire du scalp et du caractère itératif de la chirurgie.
